# Banded karyotype of Nelore cattle (*Bos taurus indicus* Linnaeus, 1758)

**DOI:** 10.3897/CompCytogen.v13i3.36449

**Published:** 2019-08-29

**Authors:** Andréia Pires Amancio, Sabrina Sara Moreira Duarte, Rafael Carneiro Silva, Alex Silva da Cruz, Danilo Conrado Silva, Claudio Carlos da Silva, Aparecido Divino da Cruz

**Affiliations:** 1 PhD Program in Biotechnology and Biodiversity, Federal University of Goiás, Rede Centro Oeste de Pós-Graduação de Pesquisa e Inovação, Rua 235, n. 40, Setor Leste Universitário, Goiânia, GO 74605-050, Brazil; 2 Replicon Research Group, Genetics Master’s Program, School of Agrarian and Biological Sciences, Pontifical Catholic University of Goiás, Rua 235, n. 40, Setor Leste Universitário, Goiânia, GO 74605-050, Brazil; 3 Genetics and Molecular Biology Master’s and PhD Program, Federal University of Goias, Avenida Esperança, s/n., Campus Samambaia, Goiânia, GO 74690-900, Brazil; 4 Human Cytogenetics and Molecular Genetics Laboratory, Health Secretary of Goias State Goiânia, GO, Brazil; 5 State University of Goiás, Campus Eseffego, Goiânia, GO, Brazil

**Keywords:** AgNOR, Brazil breeds, Cytogenetics, Karyotype, Zebu

## Abstract

Chromosome banding techniques were applied and standardized to obtain karyotype characteristics for the first time in Brazil of Nelore cattle – *Bos taurus indicus* Linnaeus, 1758 – (bovine subspecies most prominent in Brazilian livestock). Blood samples were collected from the animals of the School of Agrarian and Biological Sciences of the Pontifical Catholic University of Goiás, two males and two females of pure breed. These samples were submitted to the cell culture method to study metaphase chromosomes. Chromosome banding techniques (C, G and NOR) revealed the karyotype architecture of Nelore cattle common with that of other breeds of zebu cattle formerly karyotyped. The diploid chromosome number was invariably normal, 2n = 60. C-banding revealed C-positive heterochromatin in centromeric regions almost in all chromosomes. G-banding presented the expected band pattern in the respective chromosome pairs in correspondence with the established chromosomal patterns for the species. Ag-staining for nucleolus organizer regions (AgNOR) was identified on the telomeric end of the long arm in 7 autosomal chromosomes. In this study we found more regions in chromosomes with staining than presented in the literature for the *Bos indicus* group (BIN). These NOR regions were repeated on the same chromosomes for the 4 animals studied.

## Introduction

Nelore is an important bovine breed and well noted in Brazil for its meat production, body size and sturdiness. However, the meat industry has demanded products of higher quality. Thus, in the last 5 years, specifically with respect to meat production, the cross between Angus (taurine) and Nelore (zebu) breeds has been growing in Brazil. Indiscriminate crossing of Nelore cattle may result in a dilution of the breed and a decline in their number which may result in complete genetic extinction. Consequently, the conservation of the original breed is necessary (Reddy et al. 2016).

Despite this trend in the market, Nelore still comprises up to 80% of the national cattle of bovine breeds raised for meat, mostly due to its combination of productivity and adaptability to the tropics (Júnior et al. 2016). The states of Mato Grosso, Mato Grosso do Sul, and Goiás, a region known as Central Brazil, hold 43% of the country bovine cattle composed of Nelore breed (IBGE 2017).

Cytogenetic studies are highly useful for genetic characterization and for effective conservation of the species seriously at risk of extinction (Bharti et al. 2017). Genotype-based selection could be a powerful tool to assist farmers on making decisions regarding phenotype/genotype correlations and their interaction with the environment when managing their herds (Paulino et al. 2014).

Despite the extensive genomic investigation in cattle, not so many novelties are reported about bovine chromosomes that could be an excellent and inexpensive tool to provide important pieces of information useful for animal characterization, herd management, and evolutionary studies of breeds (David et al. 2014). The application of cytogenetic techniques has led to a simple cytological determination of the two main subspecies used in formation of domestic cattle breeds – *Bos taurus taurus* Linnaeus, 1758, Y(BTA) is submetacentric, and *Bos taurus indicus* Linnaeus, 1758, Y(BIN) is acrocentric (Halnan and Watson1982). In their karyotypes, the X chromosome is always the only morphologically distinguishable chromosome among monotonously acrocentric metaphases, being large and submetacentric (Raudsepp and Chowdhary 2016).

For correct identification of individual chromosomes, several banding techniques were developed, broadly divided into two categories: those that produce bands along the entire chromosome (Q, G, and R) and those that mark specific regions of each chromosome (C, T, or NOR) (Miranda and Mattevi 2011). Among other breeds of BIN cytogenetically studied, Nelore cattle in Brazil are still not so exploited, due to the difficulty to standardize and update the cytogentic techniques commonly used to study chromosomes, for example, the time necessary to culture cell and preparation of slides with material for banding techniques.

We are presenting in this work the necessary characterization of Nelore’s chromosomes using G-, C-, and NOR-branding methodologies.

## Material and methods

Biological samples were collected from four animals (2 male, 2 female), products industrial breeding Nelore, belonging to the study station of the Faculty of Agrarian of Biological Sciences / Pontifical Catholic University of Goiás. The herd maintained at lots of 28 m² of pasture and fed with fodder twice a day. Both males were 28 months old, weighing about 430kg. Both females were 35 months old, weighing about 480 kg. Blood samples of about 3ml of peripheral blood from the external jugular vein of each animal were kept in vaccum tubes containing heparin to prevent blood clotting and cooled on ice until arriving at the laboratory. Conventional cytological techniques were applied adapted to local and laboratory conditions of peripheral blood culturing and chromosome preparation (Verma and Babu 1995; David et al. 2014; Rosetto 2015).

### Cell culture and cytological preparation

Cell culture was performed from 1ml of blood sample transferred into RPMI 1640 (Gibco RPMI 1640 Medium) (4ml), enriched with FBS (Fetal Bovine Serum, Gibco (1ml), PHA (Phytohemagglutinin, Gibco) and antibiotics (Penicillin G sodium salt, Sigma-Aldrich) (100U/μL). The cell suspension was stored in an incubator at 38 °C under 5% of carbon dioxide (CO_2_) for 71 hours. After this time, 75μl of colchicine (Colcemid, Gibco) was added and incubation continued for an additional 30 minutes. Subsequently, samples were transferred to a 15ml conical tube and centrifuged for 10 minutes at 1000rpm, and then the supernatant was discarded (leaving about 1ml of material in the tube). A total of 10ml of hypotonic solution (KCl at 0.075 M) was added into the tube and incubated for 35 minutes at 38 °C, 5% CO_2_. The cells were then fixed with Carnoy’s solution (3 parts of methanol to 1 part of acetic acid), fixation was performed for 10 minutes at room temperature and immediately centrifuged for 10 minutes at 1000rpm. The cell pellet was fixed by three successive washes with the fixative, until the material became clear. Fixed cells were maintained in a suspension with 5ml of fixative in the refrigerator until the time of chromosomal analysis.

### C-banding

The cell suspension was dropped on a microscope slide over a water-bath steaming at 60 °C. Slides were previously cleaned and degreased to guarantee adequate spreading of metaphases. Metaphase spreads were aged in the refrigerator for 2 days. Subsequently, the slides were soaked in 0.2N HCl solution for 10 min, rinsed in distilled water. DNA denaturation was carried out in a solution of 5% barium hydroxide for 15 min at 37 °C, slides were rinsed in distilled water at room temperature. After drying, the slides were stained with 10% Giemsa’s solution for 5 minutes (KaryoMAX Giemsa Stain Solution).

### G-banding

For the GTG banding, slides with the metaphase spreads were stored at room temperature for 7 days. After aging, slides were treated in 0.025% trypsin solution (Gibco) diluted in 4mL of PBS at 37 °C for 6–7 seconds. Afterwards, slides were stained in 5% Giemsa’s solution for 5 minutes (KaryoMAX Giemsa Stain Solution).

### NOR banding

Ag-staining of NORs (Nucleolus Organizer Regions) was carried out after aging the slides for 2 days in a refrigerator. Subsequently, 2 drops of 50% silver nitrate (AgNO3, Sigma-Aldrich) and 2 drops of 2% gelatin diluted in 1% formic acid were added to the material and covered by a glass coverslip. The slide was then placed into a humid chamber at 65 °C protected from light for a time ranging from 3 to 5 minutes until the slide surface showed a copper-like color.

### Analysis of metaphases and chromosomal measurement

Metaphases were captured using white light microscopy with the aid of a karyotyping station consisting of a microscope Axioplan 2 Imaging (Carl Zeiss, Alemanha) with motorized platinum controlled by Metafer 3.4.0 software (Metasystems Corporation, Germany). Captured images were analyzed using IKAROS (Metasystems Corporation, Germany).

Twenty metaphases of each animal were analyzed. The lengths of chromosomes in micrometers were measured in mitotic metaphase of male and female cells. Karyotype symmetry/asymmetry index (S/AI), the mean length of short arm (Ls), length of long arm (Ll), total length of arm (LT), arm ratio (AR-long/short chromosome), centromeric index (CI) and type of chromosome and formula were estimated according to Eroğlu (2015).

All chromosomes measurements were translated by computation using software IKAROS (Metasystems Corporation, Germany), after pairing each pair of homologs in G- banded karyotype. Homologs were paired for all four animals, according to sex, and the final chromosome measurement corresponded to arithmetic mean of individual estimation for each chromosome.

## Results and discussion

The study of Brazilian Nelore cattle adds to the list of the zebu (*B. t. indicus*) breeds so far karylogically investigated. The diploid number in all 4 studied animals was found to be 60, consisting of 29 pairs of autosomes and one pair of sex chromosomes – the karyotype constitution, common to domestic cows of taurine/*B. taurus* and zeburine/*B. indicus* origin and established in all former reports (Wurster and Benirschke 1968; Evans et al. 1973; Mayr and Gruber 1986).

The Brazilian Nelore line originated from Ongole, a predominant breed in India (Oliveira et al. 2002). Our results were similar to those of Bharti and collaborators (2017) characterized the Ongole cattle with 29 acrocentric autosomal chromosomes and the sexual pairs, chromosome X as large submetacentric and chromosome Y as small acrocentric, thus suggesting common chromosome architecture of the Nelore cattle with that of other recognized breeds of BIN.

The measures for autosomes did not vary between male and female. Therefore, here we show the corresponding figures for the males in order to show all autosomal and both heteromorphic sex chromosome for the studied subspecies. All chromosomes measurements were represented in Table 1.

**Table 1.** The average measurements and arm ratio of the entire chromosome complement for male *Bos taurus indicus* Linnaeus, 1758, after homologs were paired up following GTG- banding.

Chromosome pair	Total length (μm)	Long arm (μm)	Short arm (μm)	Arm ritio (long/short)	Centromeric index	Chromosome type
1	67,9	61,7	6,2	9,952	9,131	A
2*	60,9	54,3	6,6	8,227	10,837	A
3*	57,8	51,8	6	8,633	10,381	A
4*	56,7	50,8	5,9	8,610	10,406	A
5	53,5	48,4	5,1	9,490	9,533	A
6	52,6	46,9	5,7	8,228	10,837	A
7	49,9	44,6	5,3	8,415	10,621	A
8	50,5	45,1	5	8,352	10,693	A
9	49,5	44,4	5,1	8,706	10,303	A
10	47,4	42,2	5,2	8,115	10,970	A
11*	45,6	40,6	5,0	8,120	10,965	A
12	42,2	37	5,2	7,115	12,322	A
13	38,7	33,1	5,6	5,911	14,470	A
14	40,3	35	5,3	6,604	13,151	A
15	38,5	33,5	5,0	6,700	12,987	A
16	38,6	33	5,6	5,893	14,508	A
17	37,8	32,2	5,6	5,750	14,815	A
18	35,6	30,2	5,4	5,593	15,169	A
19	33,5	28	5,5	5,091	16,418	A
20	32,3	26,4	6	4,475	18,266	A
21	31,7	26,5	5,2	5,096	16,404	A
22	32,2	26,9	5,3	5,075	16,460	A
23	31	26,1	4,9	5,327	15,806	A
24	29,5	24,2	5,3	4,566	17,966	A
25*	28,2	23	5,2	4,423	18,440	A
26	26,5	21,3	5,2	4,096	19,623	A
27	26,6	21,5	5,1	4,216	19,173	A
28*	25,3	20,2	5,1	3,961	20,158	A
29	22,6	18	4,6	3,913	20,354	A
X	66,6	44,2	22,4	1,973	33,634	SM
y	29,4	23,7	5,7	4,2	19,388	A

Note: A: acrocentric; SM: submetacentric. *Nucleolus organizer chromosomes.

The chromosome pairs indicate evidence of interchromosomal asymmetry. S/AI for Nelore karyotype was 2.97 and 2.98 for female and male animals, respectively, classified its karyotype between symmetric and asymmetric, most likely due to the presence of the X chromosomes. The karyotype formulae were also different for male and female Nelore cows, corresponding, respectively, to 1SM+59A and 2SM+58A. For additional discussion about the importance to know the values of the karyotype symmetry/asymmetry in higher animals, readers are strongly advised to read the work of Eroğlu (2015).

With respect to sex chromosomes in Nelore, in our results the ratio between X and Y chromosomes was 2.45 indicating a remarkable in level of allosomic heteromorphism, a common observation among animals harboring XY sex determination mechanism, leading to an evolutionary stronger reproductive isolation (Lima 2014).

Chromosome X is relatively a few larger than chromosome 1, the largest acrocentric chromosome in the bovine karyotype. X/1 proportion is close to one (1,1µm). On the other hand, Y chromosome is close in size to autosomal chromosomes 24 (BIN), with an average size of 29,5µm then compared to the smallest acrocentric chromosome 29 (BIN), Y/29 proportion was found to be 1,3. Due to its acrocentric morphology and its small size, the Y chromosome of Nelore can easily be confused with several other small autosomal chromosomes that are also acrocentric. Here we report difficulty in the identification of Y(BIN) when relying only on Giemsa staining, just as reported by Melo (2009).

However, C-, GTG-, and NOR-banding provided a better morphological characterization of all chromosomes, including Y chromosome in Nelore, facilitating the proper differentiation of autosomal and sexual chromosomes for the breed.

In our case, Y is acrocentric, as in the first descriptions of the zebu karyotype (Halnan and Watson 1982.) This decision was made based on arm ratio and centromeric index (CI) for all Y chromosomes measured. Acrocentric chromosomes generally show an extend satellite and visually may suggest the shape of submetacentric chromosomes.

**Figure 1.**
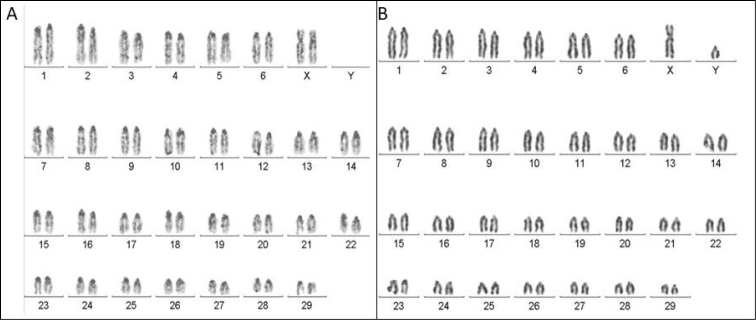
C-banded bovine chromosomes of Nelore breed. **A** female (XX) **B** male (XY).

C-banding demonstrated dark bands (C-positive) on all centromeric region of autosomes, analyzed in the bovine material which showed well-defined heterochromatin blocks. Stranzinger et al. (2007), studied the polymorphism of chromosome Y in various breeds of cattle (*Bos taurus*) in Switzerland, showed the C-negative X chromosome and C-positive Y chromosome from C-banding. However, in the animals in this study no dark bands (light or C-negative) were identified on the X and Y chromosomes (Figure 1).

The GTG banding provides alternated light and dark bands on the chromosomes, the distribution of these bands is different for each chromosome, facilitating the identification of the homologous pairs. Pinheiro et al. (1984) analyzed the BIN and BTA bovine chromosomes by G bands and found that the pattern of bands presented by the chromosomes was identical and that the difference between these animals was evidently genic.

**Figure 2.**
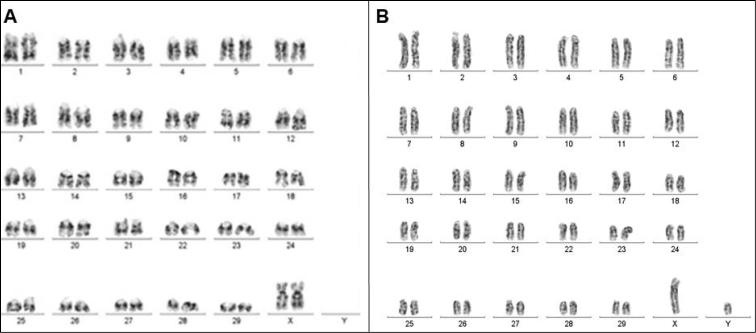
GTG-banding profile for the pairing of the chromosomes of the Nelore karyotype. **A** female (XX) **B** male (XY).

Thus, in the GTG banding analysis the haploid set of Nelore cattle consists of 29 autosomes and 1 sexual pair including X and Y chromosome. The pair composition presented in Figure 2 follows the nomenclature of the standard GTG-banded cattle karyotype (Di Berardino et al. 2001). In addition, the GTG banding can serve as a guide for the diagnosis and association of possible chromosomal alterations, being considered a differential technique for the characterization of species at chromosome levels (Rosetto 2015).

**Figure 3.**
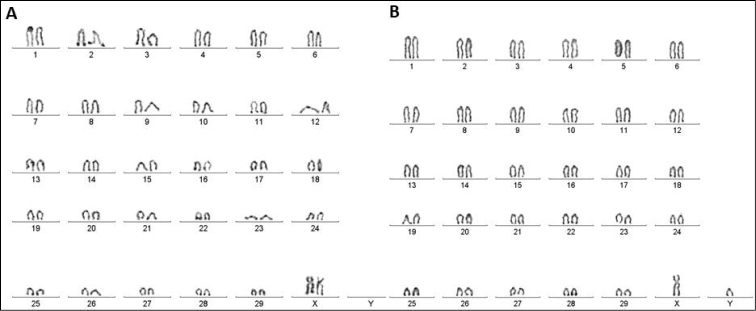
The nucleolus organizer regions on the long arm of the pairs of autosomal chromosomes 2, 3, 4, 11, 22, 25, and 28 by NOR-banding technique in female and male respectively. **A** female (XX) **B** male (XY).

In spite of the diverse qualities that the GTG- banding provides, it requires an extended time of 7 days for preparation of the slides, along with the obtaining of metaphases in good condition for the analysis of the chromosomes.

The NOR technique, initially described by Mayr and Gruber (1986), revealed 5 pairs of the zebu (*B. indicus*) chromosomes 2, 3, 4, 11 and 28 with the nucleolar organizer regions located on the long arms. That was considered an important discovery in the conserved regions in the genus *Bos* Linnaeus, 1758 and may vary within species BTA and BIN. There are genomic controversies in the literature regarding the location of the nucleolar organizer regions in the *Bos taurus* species, some breeds presented six pairs of NORs in chromosomes 2, 3, 4, 11, 25 and 28, whereas others presented 5 pairs in chromosomes 2, 3, 4, 11 and 25 respectively (Melo 2009).

The seven nucleolus organizing regions (NORs) were located on the autosomal chromosomes of cattle Nelore. The four animals that made up the sample group in this study presented the NORs in the same chromosomal pairs, which are the autosomal pairs 2, 3, 4, 11, 22, 25 and 28 shown in figure 3. In contrast, Mayr and Gruber (1986) indicated that NORs of the cattle BIN appear on eight positions of the long arm of the pair autosomes 2, 3, 4 and 28.

Jantarat and colleagues (2009) performed the banding in C, G and NOR Thai’s native cattle (*Bos taurus indicus*) and the results were compared to our study. There was a difference in the result of the NOR technique, the Thai’s native cattle presented NOR in three pairs of autosomal chromosomes whereas for the studied Nelore breed, seven pairs of chromosomes presented the silver placement in the telomeric region.

## Conclusion

About 80% of the Brazilian herd is composed of zebu breeds (*Bos t. indicus*), animals with more rusticity and easy adaptation to the predominant environment in the country (Amaral et al. 2012). Among these breeds, Nelore stands out the beef cattle with the greatest expansion in the central-west region. Therefore, it is important to study the cytogenetics of this group, being the most used chromosome banding techniques (CRPBZ 2015).

There was no cytogenetic characterization by banding techniques (C-, GTG- and NOR) for the Nelore Brazilian breed. For the animals of this study, the C banding made possible an exact identification of the acrocentric chromosomes. The technique GTG-banding provided the correct characterization of the pairs homologues, especially the autosomal chromosomes of cattle that are all acrocentric. In particular, the in this study it was possible to identify nucleolus organizing regions in other chromosomes, different from what was already known for subspecies *Bos t. indicus*.

The variation in the composition of the chromosomes that make up the national herds, especially those in this study, can be explained by the many preceding intersections and inbreeding. This management practice is commonly used to increase the herd of animals with favorable traits. Therefore, our observation can be in correspondence to the work of Carneiro et al. (2007) which refers to genetic diversity and genealogical control of the Nelore breed.

In addition to the banding techniques excellent for studies of morphology and chromosome classification, instead of new cytogenetic methodologies, such as Fluorescent In Situ Hybridization (FISH) and High Resolution Banding, can be used to understand chromosomal rearrangements and to clarify phenomena that may be related to the integrity of bovine genetic material (Luna 2012, De Lorenzi et al. 2017).

## References

[B1] Amaral G Carvalho F Capanema L Carvalho CA (2012) Panorama da pecuária sustentável. BNDS Setorial 36: 249–288.

[B2] Bharti A Panduranga Reddy P Gnana Prakash M Sakaram D (2017) Cytogenetic characterization of ongole cattle. International Journal of Advanced Biological Research 7(3): 574–577.

[B3] Carneiro TX Gonçalves EC Schneider MPC Silva A (2007) Diversidade genética e eficiência de DNA microssatélite para o controle genealógico da raça Nelore. Arquivo Brasileiro de Medicida Veterinária e Zootecnica 59(5): 1257–1262. 10.1590/S0102-09352007000500024

[B4] CRPBZ – Centro de Referencia da Pecuária Brasileira – ZEBU. Zebuinocultura 2015 http://www.zebu.org.br/Home/Secao/9331 [accessed: 10 May 2019]

[B5] David JAO Aguiar LL Mainardi VF (2014) Aplicações da citogenética em ciência animal. Deminicis BB, Martins CB (Eds) Caufes. 1ª Tópicos Especiais em Ciência Animal III. Alegre, Espirito Santo, Brasil, 222–228.

[B6] De Lorenzi L Iannuzzi A Rossi E Bonacina S Parma P (2017) Centromere Repositioning in Cattle (*Bos taurus*) Chromosome 17. Cytogenetic and Genome Research 151: 191–197. 10.1159/000473781 28494439

[B7] Di Berardino D Iannuzzi L (1981) Chromosome banding homologies in swamp and murrah buffalo. Journal of Heredity 72: 183–188. 10.1093/oxfordjournals.jhered.a109469 6168678

[B8] Di Berardino D Di Meo GP Gallagher DS Hayes H Iannuzzi L (2001) ISCNDB2000 International system for chromosome nomenclature of domestic bovids. Cytogenetics and Cell Genetics, 92: 283–299. 10.1159/000056917 11435702

[B9] Eroğlu H (2015) Which chromosomes are subtelocentric or acrocentric? A new karyotype symmetry/asymmetry index. Caryologia 68: 1–7. 10.1080/00087114.2015.1032614

[B10] Evans HJ Buckland RA Sumner AT (1973) Chromosome homology and heterochromatin in goat, sheep and ox studied by banding techniques. Chromosoma (Berlin) 42: 383–402. 10.1007/BF00399407 4730557

[B11] Halnan CRE Watson JI (1982) Y chromosome variants in cattle *Bos taurus* and *Bos indicus*. Annales de génétique et de sélection animale, INRA Editions 14(1): 1–16. 10.1186/1297-9686-14-1-1 PMC2733894 22896221

[B12] IBGE – Instituto Brasileiro de Geografia e Estatística. Diretoria de Pesquisas, Coordenação de Agropecuária, Pesquisa da Pecuária Nacional 2017 http://www.ibge.gov.br [accessed: 20 January acessado 2019]

[B13] Júnior CPB Borges LS de Sousa PHAA de Oliveira MRA Cavalcante DH de Andrade TV Barros CD Sousa Júnior SC (2016) Melhoramento Genético em Bovinos de Corte (*Bos indicus*) Efeitos ambientais, melhoramento genético animal, pecuária de corte, peso ao desmame. Nutri Time 13(1): 4558–4564.

[B14] Lima TG (2014) Higher levels of sex chromosome heteromorphism ate associated with markedly stronger reproductive isolation. Nature Communications 5(4743). 10.1038/ncomms5743 25144162

[B15] Luna HS (2012) Citogenética clássica aplicada ao monitoramento de germoplasma bovino. Revista Brasileira de Reprodução Animal, Belo Horizonte, Minas Gerais, (Brasil) 36(2): 84–93.

[B16] Mayr B Gruber K (1986) Nucleolus organizer regions and heterochromatin in the zebu (*Bos indicus* L.). Theoretical and Applied Genetics 73: 832–835. 10.1007/BF00289387 24241292

[B17] Melo TC (2009) Avaliação de aberrações cromossômicas em bovinos (*Bos taurus taurus*) infectados pelo papilomavírus bovino. Ph.D. Dissertation, Universidade Federal de Pernanbuco, Recife, Brasil.

[B18] Miranda JA Mattevi MS (2011) Técnicas de bandeamento e coloração cromossômica. Maluf SW, Riegel M Ed Artmed. Citogenética Humana, (Brasil) 63–69.

[B19] Oliveira JHR Magnabosco CU Borges AMSM (2002) Nelore: base genética e evolução seletiva no Brasil. Documentos/Embrapa Cerrados (INFOTECA-SE), Planaltina, Distrito Federal (Brasil) 49: 54 pp.

[B20] Oliveira Júnior GA Perez BC Ferraz JBS (2017) Genomics applied to puberty in beef cattle (*Bos indicus*). Revista Brasileira Reprodução Animal, Belo Horizonte, Minas Gerais (Brasil) 41(1): 264–269.

[B21] Paulino MF Detmann E Silva GA Almeida MA Márquez CED Moreno SPD Moura HF Cardenas GE Lima CAJ Martins SL Manso RM Ortega MER Lopes AS Carvalho VV (2014) Bovinocultura otimizada. 9a Simpósio internacional de produção de gado de corte, Universidade Federal de Viçosa, Viçosa, Minas Gerais (Brasil), 139–164.

[B22] Pinheiro LEL Ferrari I Ferraz JBS Almeida JR (1984) Heteromorfismo cromossômico na raça caracu. Revista Brasileira de Reprodução Animal, Belo Horizonte 8(1): 17–20.

[B23] Raudsepp T Chowdhary BP (2016) Chromosome Aberrations and Fertility Disorders in Domestic Animals. Annual Review of Animal Biosciences, 4: 15–43. 10.1146/annurev-animal-021815-111239 26884101

[B24] Reddy PRK Reddy AN Ramadevi A Kumar DS (2016) Nutritional significance of indigenous cow milk with regard to A2 beta casein – An overview. International Journal of Science, Environment and Technology 5(5) 3376–3380.

[B25] Rosetto CFR (2015) Avaliação do bandeamento cromossômico por digestão enzimática e tratamento com solução tampão citratado. Ph.D. Dissertation, Universidade Estadual Paulista Júlio de Mesquita Filho, Faculdade de Medicina de Botucatu, São Paulo.

[B26] Jantarat S Tanomtong A Kakampuy W Kaewsri S Buranarom K (2009) Standardized karyotype and idiogram of Thai’s native cattle, *Bos indicus* (Artiodactyla, Bovidae) by convention staining, G-banding, C-banding and NOR-banding techniques. Thai Journal of Genetics 2(2): 164–174. 10.14456/tjg.2009.15

[B27] Stranzinger GF Steiger D Kneubuhler J Hagger C (2007) Y chromosome polymorphism in various breeds of cattle (*Bos taurus*) in Switzerland. Journal of Applied Genetics 48: 241–245. 10.1007/BF03195218 17666776

[B28] Verma RS Babu A (1995) Human chromosomes principles and techiniques. 2^nd^ edn. McGraw-Hill, New York, 419 pp.

[B29] Wurster DH Benirschke K (1968) Chromosome studies in the superfamily Bovoidae. Chromosoma (Berlin) 25: 152–171. 10.1007/BF00327175 5709393

